# Prenatal and Postnatal Predictive Factors for Children’s Inattentive and Hyperactive Symptoms at 5 Years of Age: The Role of Early Family-related Factors

**DOI:** 10.1007/s10578-020-01057-7

**Published:** 2020-09-19

**Authors:** Hanna Huhdanpää, Isabel Morales-Muñoz, Eeva T. Aronen, Pirjo Pölkki, Outi Saarenpää-Heikkilä, Anneli Kylliäinen, E. Juulia Paavonen

**Affiliations:** 1 Department of Public Health Solutions, Finnish Institute for Health and Welfare, P.O. Box 30, 00271 Helsinki, Finland; 2 Pediatric Research Center, Child Psychiatry, University of Helsinki and Helsinki University Hospital, Helsinki, Finland; 3 Institute for Mental Health, School of Psychology, University of Birmingham, Birmingham, UK; 4 Department of Social Sciences, University of Eastern Finland, Kuopio, Finland; 5 Center for Child Health Research, University of Tampere and Tampere University Hospital, Tampere, Finland; 6 Psychology, Faculty of Social Sciences and Humanities, University of Tampere, Tampere, Finland

**Keywords:** Risk factors, Family-related factors, Inattention, Hyperactivity, Longitudinal study

## Abstract

We examined several parent-reported prenatal and postnatal factors as potential risk factors for attention-deficit and hyperactivity disorder (ADHD) symptomatology in 5-year-old children. Our study is based on the CHILD-SLEEP birth cohort. Several parental questionnaires were collected prenatally (32nd pregnancy week) and postnatally (i.e. child aged 3, 8, and 24 months and at 5 years). At 5 years of age, ADHD symptoms were assessed using questionnaires. Our main results showed that being a boy, parental depressive symptoms, more negative family atmosphere or a child’s shorter sleep duration, and maternal authoritarian parenting style predicted inattentive/hyperactive symptoms. Maternal and paternal authoritative parenting style predicted less inattentive/hyperactive symptoms. Children with several risk factors together had the highest risk for inattentive/hyperactive symptoms. Our findings emphasise the need for early screening and treatment of parental mental health, and early evidence-based targeted parental support, to enable early intervention in those children at a risk of developing ADHD.

## Introduction

Attention-deficit and hyperactivity disorder (ADHD) is the most prevalent neurodevelopmental disorder in childhood and is reported to affect approximately 5% of the population, with male predominance [1]. The core psychopathologies of ADHD are attention difficulties, impulsivity, and hyperactivity [2]. Although it is well documented that ADHD is a highly heritable disorder [3, 4], it has been estimated that 10–40% of the variance associated with ADHD is accounted for by other than genetic factors [5]. More specifically, the development of ADHD is multifactorial, including a contribution of both genetic [3] and several prenatal, perinatal, and postnatal risk factors [4–12]. Taking into account that ADHD emerges early in life and is related to a wide variety of negative physical, psychosocial, and academic outcomes [13], identifying early causal risk factors for ADHD is of crucial relevance, as this would enable early intervention in children at risk of developing ADHD [14].

Several prenatal and postnatal maternal factors have been associated with children’s elevated risk of later diagnosis of ADHD, such as a family history of ADHD [3, 9, 15], lower maternal education [15, 16], single parenthood [12, 16], maternal younger age [7, 12], mother’s prenatal and/or postnatal depression [8, 12, 17, 18], unhealthy maternal behaviours during pregnancy, including smoking [5, 6, 12, 17, 19] or alcohol use [5, 20], and premature birth/low birth weight or delivery complications [6, 7, 9, 12]. Postnatal factors such as early exposure to severe adverse life events [19, 21], harsh-intrusive/hostile parenting style [9, 22, 23], and exposure to certain chemicals (such as lead, phthalates, and organophosphate pesticides) [19] have been also related to difficulties in symptoms of inattention and/or hyperactivity later in childhood. Finally, according to a previous meta-analysis, children’s sleeping difficulties, and especially shorter sleep duration, is associated with symptoms of inattention/hyperactivity [24], both in cross-sectional [25] and longitudinal studies [10, 26]. Shorter sleep duration during infancy has been linked to later ADHD-related symptoms [10, 26]. Most of the previous studies of early risk factors and ADHD have, however, typically focused on isolated risk factors, only considering a limited amount of the aforementioned potential risk factors. Previous reviews suggest a need for new studies that include a wider range of potential risk factors acting together during pregnancy, infancy, and early childhood, and provide a more comprehensive picture of the risk factors related to ADHD [4, 11].

The disruptive nature of a child’s ADHD symptoms can influence several aspects of family functioning. It has been well-documented that the presence of ADHD in children is associated with varying degrees of disturbances in marital functioning, more conflicted family environment, poorer parent–child relationships, more parenting stress, and increased parental psychopathology [27, 28]. Cross-sectional studies and a meta-analysis have demonstrated that parents of children with ADHD exhibit higher levels of depression, anxiety, and parenting stress compared to healthy children [28–30]. Furthermore, the presence of parental psychopathology may negatively influence the parents’ ability to supportively respond to their child’s ADHD symptoms [30]. Moreover, parents of children with ADHD report more inconsistent and hostile parenting behaviours, and less parental warmth and sensitive behaviour toward their children compared to typically developing children [29, 31–33]. Poorer parenting skills in these families may contribute to the development of additional behavioural problems that worsen ADHD outcomes [30, 31]. Most of the previous studies of parenting and ADHD are, however, cross-sectional in nature and focus on children who already have a diagnosis.

Surprisingly, little longitudinal research has been done on the family-related factors of early life, such as parental mental health, family atmosphere, and/or parenting behaviours, and their contribution to the developmental course of ADHD symptoms [34]. It has been suggested that a child’s ADHD symptoms negatively affect the mother–child relationship and increase the use of negative parenting strategies (e.g. mother–child hostility), rather than negative parenting having a causal role in ADHD [35, 36]. For instance, a large Canadian population-based birth cohort study (N = 2057 children) noted that parent-reported family dysfunction or poorer parenting strategies at the age of 5 months did not predict higher levels of inattentive/hyperactive symptoms in school-age children [12]. Furthermore, a longitudinal twin study with school-aged children and adolescents showed that associations between parent–child-hostility and ADHD symptoms appeared to largely be accounted for by genetic factors [35]. For instance, while boys’ ADHD symptoms had an impact on mother–child-hostility, there were no effects in the opposite direction.

Nevertheless, previous research on early parenting and later symptoms of inattention/hyperactivity have also reported contradictory findings [9, 22, 23, 37–40]. These studies suggest that poorer parenting in early childhood may serve as a risk factor that moderates and exacerbates a child’s predisposition to present symptoms of inattention and hyperactivity. For instance, early intrusive (controlling/parent-agenda driven behaviours) and/or negative (hostile verbal and physical punishment) parenting when the child is 6-months-old has been associated with inattentive/hyperactive symptoms at preschool [9, 23] and school age [9, 22]. Furthermore, maternal sensitive parenting during infancy and early preschool age has been associated with the development of inhibitory [38] and attentional [40] control at preschool age and fewer ADHD or oppositional defiant disorder (ODD) symptoms at school age [37]. One longitudinal study has also found an indirect pathway in which parenting hostility mediated the relationship between maternal postnatal mental health problems and children’s ADHD symptoms at school age [39]. Most of the previous studies on the association between early parenting and later ADHD-symptoms have not, however, controlled for other well-known prenatal and postnatal risk factors, including for example low birth weight, smoking/alcohol use during pregnancy, socioeconomical factors, parental ADHD symptoms or parental prenatal/postnatal depressive symptoms.

Until recently, there has been a lack of longitudinal studies that consider a broad range of both parental and early childhood factors as moderating the risk for ADHD symptoms at preschool age [12]. Therefore, the aim of our study was to identify which prenatal and postnatal parent-reported risk factors predict inattentive and/or hyperactive symptoms at 5 years old. Based on previous studies [4–12, 17, 22], we hypothesised that maternal depression during and after pregnancy, children’s sleep duration, a hostile, punitive, and nonresponsive (authoritarian) parenting style, children’s low birth weight, low parental education, parental ADHD, low income, and maternal smoking and alcohol use during pregnancy would predict symptoms of inattention and hyperactivity at 5 years old. Furthermore, we hypothesised that a maternal consistent, supportive, and warm (authoritative) parenting style would predict less inattentive/hyperactive symptoms [37, 38, 40].

## Methods

### Participants

This study is based on the population-based CHILD-SLEEP birth cohort in Finland, with several measurement points from pregnancy until the children were 5 years old [41]. More specifically, the recruitment and baseline measurement occurred prenatally at the 32nd week, with the follow-up measurements taking place at childbirth and at the ages of 3, 8, 18, and 24 months and at 5 years. A total of 2,244 parents consented to receive the prenatal questionnaires during their visits to the maternity clinics, of which 1,679 (74.8%) families gave their consent to participate in the study and returned the baseline questionnaires. For this study, we used data from the parental questionnaires completed at pregnancy (week 32) and when the children reached the ages of 3, 8, and 24 months and 5 years. Prenatally, detailed information on parental sociodemographic and health factors was collected. Furthermore, parents estimated the family atmosphere and the duration of their child’s sleep at the age of 3 months, parenting style at 8 months, and the child’s inattentive and hyperactive symptoms at 5 years old.

The response rate at 5 years old was 42.5% (N = 714). Furthermore, we excluded cases with severe chronic illnesses or disabilities, such as Down’s syndrome or Hirschsprung disease (n = 7), and all twins (n = 8). Thereby, the final sample consisted of 699 children for whom either the Strengths and Difficulties Questionnaire (SDQ) or the Five-to-Fifteen (FTF) questionnaire was completed at 5 years old. The SDQ was available for 666 children and the FTF questionnaire for 671 children, while both SDQ and FTF questionnaires were available for 638 children. In a majority of these cases (n = 699), SDQ and FTF questionnaires were filled by mothers (n = 487). Some parents filled the questionnaire together (n = 170). Of the final sample (n = 699), prenatal questionnaires were available for 697 mothers and 670 fathers. At 3 months old, the parental responses regarding their child’s sleep duration were available for 654 families, and on family atmosphere for 671 mothers and 641 fathers. At 8 months old, the parental responses on parenting style were available for 674 mothers and 621 fathers, respectively. Finally, the parental depression trajectory, which was obtained from a depression questionnaire measured prenatally and again when the child was aged 3, 8, and 24 months, was available for 698 mothers and 674 fathers.

The respondents at 5 years old (n = 699) differed from the nonresponding parents in some demographic characteristics. For instance, the responding parents had a higher educational level (*p* < 0.05) and the number of children in the family during pregnancy was lower (0.71 compared with 0.80; p < 0.05). Furthermore, the responding mothers were slightly older (31.2 years compared with 30.3 years; *p* ≤ 0.001) and had fewer symptoms of ADHD (Adult ADHD Self-Report Scale ASRS total score 1.03 compared to 1.31; *p* ≤ 0.001). The responding fathers reported less current smoking during pregnancy (p < 0.01) and had more depressive symptoms (p < 0.05). There were, however, no differences in sex, birth weight, gestational age, maternal depressive symptoms, paternal ADHD symptoms or maternal smoking during pregnancy.

The ethics committee of Pirkanmaa Hospital District approved the study protocol (R11032) and all participants with a written informed consent were eligible for the study.

## Measures

### Outcome

Inattentive and hyperactive symptoms at 5 years of age were assessed using two different parent-reported questionnaires: the FTF [42], and the SDQ [43]. The FTF questionnaire comprises 181 statements with three response alternatives for 5–15-year-olds, related to behavioural or developmental problems. In this study, we used 18 items reflecting the same symptoms as found in the DSM-IV criteria for ADHD, comprising the 9-item inattention domain and the 9-item hyperactivity-impulsivity domain. The FTF inattention total score was the sum of the 9 inattention items, and the FTF hyperactivity-impulsivity total score was the sum of the 9 hyperactivity-impulsivity items. The cut-off points for both FTF inattention and hyperactivity-impulsivity domains were 6 points or more, corresponding to the 75th percentile in our 5-year-old sample (Table 1). Children scoring in the 75th percentile or over in FTF inattention and/or hyperactivity scales were considered to have inattentive and/or hyperactive symptoms. This specific 75th percentile cut off criteria was considered to allow sufficient sample size in each category.

**Table 1:** Means (SD) and frequencies (%) of the child’s variables of interest

	Children (N = 699)	
	N (%)	Mean (SD)
Sex (male/female)	366 (52.4) / 333 (47.6)	–
Age at 5 years (months)	642	68.0 (5.1)
Birth weight (g)	658	3586 (451)
Gestational weeks	681	40.0 (1.2)
Apgar score	547	8.4 (1.1)
Apgar score < 7	23 (3.3)	–
Preterm birth (< 37 weeks)	11 (1.6)	–
Short sleep duration (< 13.0 h) at 3 months	171 (26.7)	–
SDQ Hyperactivity score at 5 years	666	3.1 (2.3)
FTF Inattention score at 5 years	668	3.8 (3.4)
FTF Hyperactivity-Impulsivity score at 5 years	670	4.0 (3.6)
SDQ Hyperactivity score > 75pc at 5 years	171 (25.7)	–
FTF Inattention score > 75pc at 5 years	174 (26.0)	–
FTF Hyperactivity-Impulsivity score > 75pc at 5 years	187 (27.9)	–

*SDQ* Strengths and Difficulties Questionnaire, *FTF* Five- to Fifteen Questionnaire, *pc* percentile

The SDQ is a brief child psychiatric screening questionnaire for 3–16-year-olds and includes 25 items. Parents rate the statement best describing their child’s behaviour on a 3-point scale. In this study, we only used the 5-item inattention-hyperactivity scale. The total scale score was the sum of 3 items and 2 reversed items. The cut-off point for inattentive/ hyperactive symptoms was 5 points or more, corresponding to the 75th percentile of the 5-year-old children in this study. Children scoring in the 75th percentile or over on the SDQ inattention-hyperactivity scale were considered to have inattentive/hyperactive symptoms.

### Early Risk Factors

Background information including parental age, number of previous children, parental net income, and parental education was included on the prenatal questionnaires. Parental educational level was coded as 1 = “none or some vocational training”, 2 = “vocational training or some further education colleges, and 3 = “university”. Parental low income was defined as having a personal net income below 1,000 euros per month (no/yes). For mothers, smoking during pregnancy (no/yes) referred to having smoked at least once during the past six months. For fathers, smoking during pregnancy (no/yes) referred to current smoking. Maternal alcohol consumption during pregnancy was coded as 1 = yes, if alcohol was consumed at least once monthly, whereas for fathers, alcohol needed to have been consumed at least twice a week to be coded as 1 = yes.

Maternal and paternal depressive symptoms were measured prenatally and again when the child was aged 3, 8, and 24 months using the 10-item version of the Center for Epidemiological Studies Depression Scale (CES-D) [44, 45]. Caregivers were asked to rate how often over the past week they have experienced depressive symptoms, such as feeling lonely, feeling depressed, and restless sleep. The items were rated on a four-point scale and the total score was the sum of 8 items and 2 reversed items, with a higher score indicating more severe depressive symptoms (scale range 0–30 points). Latent profile analysis was used to construct a trajectory of maternal and paternal depressive symptoms from pregnancy to 2-years-old. For both parents, a solution with three stable depressive symptom trajectories (persistent low, moderate, or high levels of symptoms) was considered the best fitting and most informative. A detailed description of the parental trajectories is available elsewhere [46]. Finally, the maternal depressive symptom trajectory was dichotomized as 1 = persistent low levels of depressive symptoms and 2 = persistent moderate or high depressive symptoms (see Table 4a-b).

The maternal and paternal ADHD symptoms were measured prenatally using a six-item version of the Adult ADHD Self-Report Scale (ASRS) [47]. ASRS includes questions about the frequency of any recent DSM-IV criterion for adult ADHD. Parents evaluated how often they had hyperactive/inattentive symptoms on a five-point scale (i.e. 0 = “never”, 1 = “seldom”, 2 = “sometimes”, 3 = “often”, and 4 = “very often”). Items 1–3 were recoded as a dichotomous variable indicating a problem: 0 = “never/seldom”, and 1 = “sometimes to very often”. Items 4–6 were recoded as a variable indicating a problem: 0 = “never to sometimes”, and 1 = “often or very often”. The total score was the sum of the six dichotomous items. An ASRS total score of < 4 indicated “no ADHD symptoms” and an ASRS score of ≥ 4 indicated the presence of “ADHD symptoms”.

Mothers and fathers were asked to evaluate the social relationships and marital dissatisfaction in their family by using a family atmosphere scale [41] when the child was 3 months old. This scale includes seven items rated on a seven-point semantic differential scale (e.g., 1 = “approving”—7 = “disapproving”; 1 = “safe” – 7 = “unsafe”; “1 = “quarrelsome” – 7 = “harmonious”). Three of the items were reverse-coded and a summary score was calculated. All seven items loaded one factor indicating one-dimensionality of the measure. The Cronbach’s Alphas were 0.86 and 0.87 for maternal and paternal scales, respectively. For the purpose of this study, a good family atmosphere was defined using the 75th percentile of the summary score (sum score ≥ 46 for mothers; sum score ≥ 45 for fathers), while the families with more negative family atmosphere consisted of those having a summary score < 75th percentile.

Mothers and fathers were asked to assess their parenting style when their child was 8 months old by using a 38-item version of the Parenting Styles and Dimensions Questionnaire (PSDQ) [48]. The PSDQ includes three global parenting dimensions consistent with Baumrind’s authoritarian (i.e. marked by verbal hostility, punishment, punitive strategies, directiveness, and low levels of emotional support and responsiveness), authoritative (i.e. marked by high levels of emotional support and responsiveness, parental warmth and involvement, reasoning, democratic participation and patience with a child), and permissive (i.e. marked by lack of consistency, ignoring the child’s misbehaviour, and parental uncertainty about parenting abilities) parenting styles [49]. Parents were asked to evaluate how often they exhibit a behaviour on a five-point scale (i.e. 1 = “never”, 2 = “once in a while”, 3 = “about half of the time”, 4 = “very often” and 5 = “always”). For this study, 24 items were excluded from the original 62-item version, as the excluded items referred to significantly older (preschool/school-aged) children. Of the 38 remaining items, 13 reflected authoritative, 12 authoritarian, and 13 permissive parenting styles. Three of the permissive parenting items were reverse-scored. Summary scores for mothers and fathers were separately calculated for each parenting style. Cronbach’s Alphas were calculated for each summary score: (a) authoritarian parenting α = 0.74 for mothers, and α = 0.76 for fathers; (b) authoritative parenting α = 0.79 for mothers, and α = 0.83 for fathers; and (c) permissive parenting α = 0.53 for both parents. The cut-off points of each summary score, which corresponded to the 75th percentile, were: (a) authoritarian parenting style: sum score ≥ 22.0 for mothers and fathers; (b) authoritative parenting style: sum score ≥ 58.5 for mothers, and sum score ≥ 56.7 for fathers; and (c) permissive parenting style: sum score ≥ 29.3 for mothers, and sum score ≥ 27.1 for fathers. Scores over the 75th percentile indicated “authoritarian parenting”, “authoritative parenting”, and “permissive parenting”, respectively. Finally, to compare the prevalence of the elevated inattentive and/or hyperactive symptoms measured by SDQ and FTF between children with and without an authoritarian parent, a new variable including the following categories was created: 0 = “no maternal or paternal authoritarian parenting”, 1 = “maternal authoritarian parenting, no paternal authoritarian parenting”, 2 = “paternal authoritarian parenting, no maternal authoritarian parenting”, and 3 = “maternal and paternal authoritarian parenting”. A similar variable was created also for authoritative parenting.

The children’s sleep duration at the age of 3 months was measured using the Brief Infant Sleep Questionnaire [50]. For this study, we selected the items of night-time sleep and daytime sleep in hours. The total sleep duration was calculated as the sum of daytime and night-time sleep in hours per day. Extreme outliers (sleep duration < 7.0 h or 20.0 > hours) were excluded (n = 14). Short sleep duration (< 13.0 h per day) was defined using the 25th percentile, based on the sample of this study.

### Missing Values

The missing answers in the subscales (Family atmosphere, PSDQ, SDQ, and FTF) were imputed by the individual mean if at least 60% of the answers were available. Otherwise, the subscale score was considered missing. The missing values were, however, infrequent in the data set. Approximately, 2–5% of answers per item in the family atmosphere or PSDQ subscales were missing. At the age of *5* years, approximately 2% of the answers were missing. Missing values in the background factors such as birth weight (N = 41), number of children prenatally (N = 58), mother’s (N = 9) or father’s (N = 84) age, educational level of mothers (N = 3) or fathers (N = 34), and mother’s (N = 14) or father’s (N = 40) net income per month were imputed by the item mean. The analyses were conducted with both the imputed and not-imputed datasets. Sensitivity analysis showed that the results remained similar in both regards. Thus, in this study, we only report the results of the imputed datasets.

### Statistical Analysis

All analyses were performed using IBM SPSS Statistics 25. Firstly, the distribution of the variables of interest was described. Secondly, multinomial logistic regression analyses were used to assess whether several prenatal and postnatal factors predict a child’s inattentive/hyperactive symptoms at 5 years old. The outcome variables (FTF inattention, FTF hyperactivity-impulsivity, and SDQ inattention-hyperactivity scale scores) were dichotomous yes/no (children with inattentive/hyperactive symptoms, scale scores ≥ 75th percentile), and each outcome variable was examined in different models. The explanatory variables included continuous (child aged 5 years, birth weight, number of children in the family and parental age prenatally), categorical (parental education, and parental depressive symptoms), and dichotomous (gender, short sleep duration, low parental income, parental alcohol/tobacco use during pregnancy, parental prenatal ADHD symptoms, good family atmosphere, and PSDQ parenting styles) variables.

Univariate analyses were first done with one explanatory factor at a time in the model. Next, to find out the best combination of explanatory factors to predict the child’s inattentive/hyperactive symptoms at 5 years, multivariate analyses were performed using the backward stepwise selection method, which begins with all predictors in the initial (full) model and then eliminates variables in successive steps until no variables can be removed without statistically significant loss of model fit.

Third, the prevalence of inattentive/hyperactive symptoms at 5 years old (scoring over 75th percentile in SDQ/FTF inattentive/hyperactive scale score) in different parenting groups (authoritarian and authoritative parenting styles) were compared using χ^2^ tests.

Finally, to examine the cumulative effect of the three most significant risk factors in the predictive multivariate models, we studied the odds ratios (OR) related to different risk factor combinations relative to the no risk factors status (i.e. the children without any risk factors for inattentive/hyperactive symptoms measured by SDQ were separately compared to children with one or more risk factors). We reported the odds of developing inattentive/hyperactive symptoms given each combination of risk factors relative to the odds of developing inattentive/hyperactive symptoms given the non-exposure risk factor status. Next, the ORs for different risk factor combinations were summarised in Table 4a. Finally, in a separate analysis, we only selected all the boys from our sample at 5 years in order to examine the cumulative effect of these three risk factors in boys. This was done because we found that being a boy predicted ADHD symptoms in all our multinomial logistic regression models (Table 4b).

## Results

Descriptive statistics on the children and their families are reported in Tables 1–2. Of the sample, 48.2% of the cases were females and the vast majority of the children were born full-term, with only 1.6% being born before the 37th gestational week (which was due to the inclusion criteria of the study). The mean 1 min Apgar score was 8.4, and 3.3% had Apgar score < 7, reflecting low levels of possible delivery complications.

**Table 2:** Means (SD) and frequencies of the parental variables of interest

	*Mothers (N* = *697)*		*Fathers (N* = *674)*	
	*N*^*c*^	*Mean (SD) / N (%)*	*N*^*c*^	*Mean (SD) / N (%)*
Parental age during pregnancy	690	31.2 (4.59)	615	32.8 (5.11)
Number of children in the family during pregnancy	641	0.71 (0.88)	641	0.71 (0.88)
Low parental income < 1000 €/month during pregnancy	685	140 (20.4%)	659	45 (6.8%)
Parental Education during pregnancy	681	–	665	–
None or some vocational training		31 (4.6%)		63 (9.6%)
Vocational degree or further education colleges		401 (58.9%)		378 (57.6%)
University		249 (36.6%)		215 (32.8%)
Alcohol use during pregnancy (yes)^a^	690	102 (14.8%)	665	200 (30.1%)
Never drinking ≥ 6 doses per time	567	560 (98.8%)	658	92 (14.0%)
Tobacco use during the pregnancy^b^	694	33 (4.7%)	96	96 (14.4%)
Depressive symptoms (CES-D)	698	–	674	–
No depression		453 (64.9%)		485 (72.0%)
Moderate		193 (27.7%)		176 (26.1%)
High		52 (7.4%)		13 (1.9%)
ADHD symptoms (ASRS > 4) during pregnancy	693	23 (3.3%)	664	48 (7.2%)
Parental ADHD diagnosis	628	3 (0.5%)	587	2 (0.3%)
Parental divorce during the past 5 years		55(7.9%)		22 (3.1%)
Good Family Atmosphere at 3 months > 75pc	669	149 (22.3%)	640	173 (27.0%)
Parenting style (PSDQ) at 8 months				
Authoritarian parenting > 75pc	672	166 (24.7%)	615	136 (22.1%)
Authoritative parenting > 75pc	674	169 (25.1%)	621	160 (25.8%)
Permissive parenting > 75pc	671	152 (22.7%)	617	177 (28.7%)

^a^For mothers: having smoked at least once during the past six months; for fathers: current smoking

^b^For mothers: alcohol use at least monthly during pregnancy; for fathers: at least twice a week

^c^n refer to available data for the specific measure

*CES-D* Center for Epidemiological Studies Depression Scale, *ASRS* Adult ADHD Self Report Scale, *PSDQ* Parenting Styles and Dimensions Questionnaire, *pc* percentile

Our results from the univariate and multinomial regression models are reported in Table 3. First, univariate analyses showed, that gender (being a boy) was associated with ADHD-related symptoms at 5 years of age. Further, moderate and high maternal depression levels and more negative family atmosphere at the age of 3 months were associated with more inattentive/hyperactive symptoms. Furthermore, a maternal authoritarian parenting style when the child was 8 months old was associated with ADHD-related symptoms, and authoritative parenting style was associated with less inattentive/hyperactive symptoms. Children’s shorter sleep duration at the age of 3 months was related to inattentive symptoms measured by FTF in 5-year-old children. Finally, maternal advanced age and paternal moderate depressive levels were related to hyperactive/impulsive symptoms.

**Table 3:** A-C Parent-reported prenatal and postnatal predictors of hyperactive and inattentive symptoms in 5-year-old children

A. SDQ Inattention-Hyperactivity > 75pc (N = 171)	Mothers (N = 583)^a^						Fathers (N = 528)^a^					
	Univariate			Multivariate^b^			Univariate			Multivariate^b^		
	*OR*	*CI 95%*	*p*	*OR*	*CI 95%*	*p*	*OR*	*CI 95%*	*p*	*OR*	*CI 95%*	*p*
Gender (being a boy)	**1.82**	**1.27–2.61**	**0.001**	**1.87**	**1.25–2.79**	**0.002**	**1.82**	**1.27–2.61**	**0.001**	**1.75**	**1.17–2.64**	**0.007**
Birth weight	1.00	0.97–1.04	0.894				1.00	1.00–1.00	0.965			
Child’s age at 5 years old	1.01	0.97–1.04	0.726				1.01	0.97–1.04	0.726			
Number of children in the family during pregnancy	1.18	0.97–1.44	0.105				1.18	0.97–1.44	0.105			
Child’s short sleep duration at 3 months < 13 h	1.03	0.69–1.55	0.883				1.03	0.69–1.55	0.883			
Parental age during pregnancy	1.00	0.96–1.04	0.835				1.02	0.98–1.06	0.358			
Low parental income < 1000 €/month during pregnancy	1.09	0.71–1.67	0.689				2.35	0.98–5.66	0.056	**3.01**	**1.03–8.78**	**0.044**
Parental Education during pregnancy			0.128			0.070			0.612			
None or some vocational training												
Vocational degree or further education colleges	0.64	0.30–1.40	0.264	0.90	0.38–2.12	0.805	1.03	0.56–1.91	0.917			
University	0.48	0.22–1.08	0.076	0.56	0.23–1.34	0.194	0.84	0.44–1.64	0.621			
Alcohol use during pregnancy (yes/no)^c^	0.98	0.60–1.62	0.949				1.09	0.74–1.60	0.677			
Tobacco use during the pregnancy^d^	1.79	0.86–3.74	0.123				1.49	0.93–2.40	0.099			
Depressive symptoms (CES-D) No depression			**0.001**			**0.046**			0.282			
Moderate	**1.49**	**1.01–2.20**	**0.046**	1.30	0.83–2.02	0.247	1.27	0.85–1.89	0.245			
High	**3.05**	**1.67–5.57**	**0.000**	**2.39**	**1.18–4.84**	**0.016**	2.01	0.64–6.26	0.231			
ADHD symptoms (ASRS > 4) during pregnancy	2.04	0.86–4.86	0.108				1.32	0.69–2.54	0.404			
More negative family atmosphere at 3 months^e^	**2.22**	**1.35–3.63**	**0.002**	**1.81**	**1.05–3.13**	**0.034**	**1.74**	**1.12–2.72**	**0.015**	1.51	0.93–2.46	0.097
Parenting style (PSDQ) at 8 months												
Authoritarian parenting at 8 months > 75pc	**2.51**	**1.70–3.71**	**0.000**	**2.14**	**1.39–3.33**	**0.001**	1.47	0.96–2.26	0.076			
Authoritative parenting at 8 months < 75pc	**1.59**	**1.03–2.44**	**0.038**				**1.78**	**1.12–2.81**	**0.014**	**1.73**	**1.04–2.88**	**0.036**
Permissive parenting at 8 months > 75pc	1.19	0.79–1.81	0.402				1.21	0.81–1.81	0.362			

*B.FTF Inattention* > *75pc (N* = *174)*	*Mothers (N* = *586)*^*a*^						*Fathers (N* = *538)*^*a*^					
	*Univariate*			*Multivariate*^*b*^			*Univariate*			*Multivariate*^*b*^		
	*OR*	*CI 95%*	*p*	*OR*	*CI 95%*	*p*	*OR*	*CI 95%*	*P*	*OR*	*CI 95%*	*p*
Gender (being a boy)	**1.94**	**1.36–2.78**	**0.000**	**1.87**	**1.27–2.77**	**0.002**	**1.94**	**1.36–2.78**	**0.000**	**1.83**	**1.22–2.74**	**0.003**
Birth weight	1.00	0.96–1.03	0.892				1.00	0.99–1.00	0.309			
Child’s age at 5 years old	1.01	0.97–1.04	0.706				1.01	0.97–1.04	0.706			
Number of children in the family during pregnancy	0.82	0.65–1.03	0.082	0.78	0.60–1.01	0.064	0.82	0.65–1.03	0.082			
Child’s short sleep duration at 3 months < 13 h	**1.91**	**1.30–2.82**	**0.001**	**1.87**	**1.23–2.84**	**0.003**	**1.91**	**1.30–2.82**	**0.001**	**1.87**	**1.22–2.87**	**0.004**
Parental age during pregnancy	1.01	0.97–1.04	0.715				1.03	0.99–1.07	0.120			
Low parental income < 1000 €/month during pregnancy	0.86	0.57–1.32	0.496				0.97	0.49–1.92	0.922			
Parental Education during pregnancy None or some vocational training			0.852						0.664			
Vocational degree or further education colleges	0.96	0.41–2.22	0.917				0.96	0.52–1.74	0.881			
University	0.87	0.36–2.06	0.743				0.81	0.43–1.54	0.518			
Alcohol use during pregnancy (yes/no)^c^	0.97	0.60–1.59	0.915				0.87	0.59–1.29	0.498			
Tobacco use during the pregnancy^d^	1.38	0.64–3.00	0.413				0.87	0.52–1.45	0.593			
Depressive symptoms (CES-D) No depression			**0.044**						**0.043**			**0.049**
Moderate	1.40	0.95–2.06	0.089				1.46	0.99–2.16	0.058	1.11	0.71–1.74	0.644
High	**1.98**	**1.06–3.69**	**0.033**				2.82	0.93–8.56	0.068	**5.13**	**1.38–19.0**	**0.015**
ADHD symptoms (ASRS > 4) during pregnancy	1.64	0.67–3.97	0.277				1.24	0.64–2.38	0.515			
More negative family atmosphere at 3 months^e^	**2.04**	**1.27–3.31**	**0.003**	**1.82**	**1.08–3.06**	**0.025**	1.54	0.99–2.39	0.053			
Parenting style (PSDQ) at 8 months												
Authoritarian parenting > 75pc (PSDQ)	1.41	0.95–2.10	0.087	**1.64**	**1.04–2.59**	**0.034**	0.99	0.63–1.54	0.949			
Authoritative parenting < 75pc (PSDQ)	1.48	0.96–2.26	0.073				1.50	0.96–2.34	0.075	1.54	0.95–2.51	0.079
Permissive parenting > 75pc (PSDQ)	0.99	0.64–1.51	0.960				0.86	0.57–1.30	0.472			

*C. FTF Hyperactivity-Impulsivity* > *75pc (N* = *187)*	*Mothers (N* = *588)*^*a*^						*Fathers (N* = *540)*^*a*^					
	*Univariate*			*Multivariate*^*b*^			*Univariate*			*Multivariate*^*b*^		
	*OR*	*CI 95%*	*p*	*OR*	*CI 95%*	*p*	*OR*	*CI 95%*	*p*	*OR*	*CI 95%*	*p*
Gender (being a boy)	**1.76**	**1.24–2.49**	**0.001**	**1.82**	**1.23–2.68**	**0.003**	**1.76**	**1.24–2.49**	**0.001**	**1.70**	**1.12–2.56**	**0.012**
Birth weight	1.01	0.98–1.04	0.599				1.00	0.99–1.00	0.305			
Child’s age at 5 years old	1.01	0.98–1.04	0.414				1.01	0.98–1.04	0.414			
Number of children in the family during pregnancy	1.01	0.82–1.24	0.904				1.01	0.82–1.24	0.904			
Child’s short sleep duration at 3 months < 13 h	1.11	0.75–1.64	0.599				1.11	0.75–1.64	0.599			
Parental age during pregnancy	**1.04**	**1.00–1.08**	**0.034**	**1.06**	**1.02–1.10**	**0.007**	1.12	0.98–1.05	0.327			
Low parental income < 1000 €/month during pregnancy	0.90	0.59–1.36	0.618				2.20	0.96–5.00	0.062	**9.30**	**2.12–40.8**	**0.003**
Parental Education during pregnancy			0.241						0.944			
None or some vocational training												
Vocational degree or further education colleges	1.77	0.66–4.74	0.260				1.09	0.59–2.00	0.787			
University	2.14	0.79–5.83	0.136				1.12	0.59–2.13	0.735			
Alcohol use during pregnancy (yes/no)^c^	1.13	0.71–1.81	0.604				1.22	0.84–1.77	0.289	1.47	0.96–2.23	0.074
Tobacco use during the pregnancy^d^	0.60	0.24–1.49	0.271				0.82	0.50–1.36	0.446			
Depressive symptoms (CES-D) No depression			**0.028**			**0.005**			**0.037**			**0.022**
Moderate	1.36	0.93–2.00	0.110	1.48	0.97–2.24	0.066	**1.51**	**1.03–2.22**	**0.033**	1.27	0.82–1.98	0.288
High	**2.14**	**1.16–3.94**	**0.015**	**2.95**	**1.45–6.00**	**0.003**	2.54	0.84–7.74	0.099	**7.31**	**1.66–32.2**	**0.009**
ADHD symptoms (ASRS > 4) during pregnancy	2.19	0.93–5.17	0.072				0.87	0.44–1.72	0.689			
More negative family atmosphere at 3 months^e^	**1.73**	**1.10–2.708**	**0.017**				1.42	0.93–2.16	0.102			
Parenting style (PSDQ) at 8 months												
Authoritarian parenting > 75pc	**1.73**	**1.18–2.54**	**0.005**				0.94	0.61–1.45	0.779			
Authoritative parenting < 75pc	**1.93**	**1.25–2.99**	**0.003**	**1.84**	**1.14–2.94**	**0.012**	**2.17**	**1.37–3.44**	**0.001**	**2.47**	**1.49–4.10**	**0.000**
Permissive Parenting > 75pc	0.98	0.64–1.47	0.903				1.12	0.75–1.66	0.581			

Bold values denote statistical significance at the p < 0.05

TF Hyperactivity-Impulsivity domain was available for 670 children

Multivariate model shows the odds ratios (OR) from multinomial regression analyses

SDQ Inattention-Hyperactivity domain was available for 666 children

^a^Prenatal information for mothers (n = 697) and prenatal information for fathers (n = 674) was used as a base category

^b^Using backward stepwise selection method

^c^For mothers: having smoked at least once during the past six months; for fathers: current smoking

^d^For mothers: alcohol use at least monthly during pregnancy; for fathers: at least two times a week

^e^Family atmosphere summary score < 75th percentile

*SDQ* Strengths and Difficulties questionnaire, *CES-D* Center for Epidemiological Studies Depression Scale, *ASRS* Adult ADHD Self Report Scale, *PSDQ* Parenting Styles and Dimensions Questionnaire, *pc* percentile, *FTF* Five-To-Fifteen Questionnaire

Second, in multinomial regression models, high maternal and paternal depression levels were associated with more inattentive/hyperactive symptoms. Paternal low income was also associated with inattentive/hyperactive symptoms. Parental prenatal ADHD symptoms (ASRS score > 4) were not, however, related to the child’s inattentive/hyperactive symptoms. Children’s shorter sleep duration was associated with inattentive symptoms.

In addition, a maternal authoritarian parenting style was related to more inattentive/hyperactive symptoms. Furthermore, the maternal authoritative parenting style predicted less hyperactive symptoms measured by FTF, and good family atmosphere when the child was 3 months old reported by the mother was also associated with lower risk for inattentive/hyperactive symptoms. The paternal authoritative parenting style predicted less inattentive/hyperactive symptoms. The permissive parenting style was not related to any ADHD-related symptoms.

**Fig. 1:**
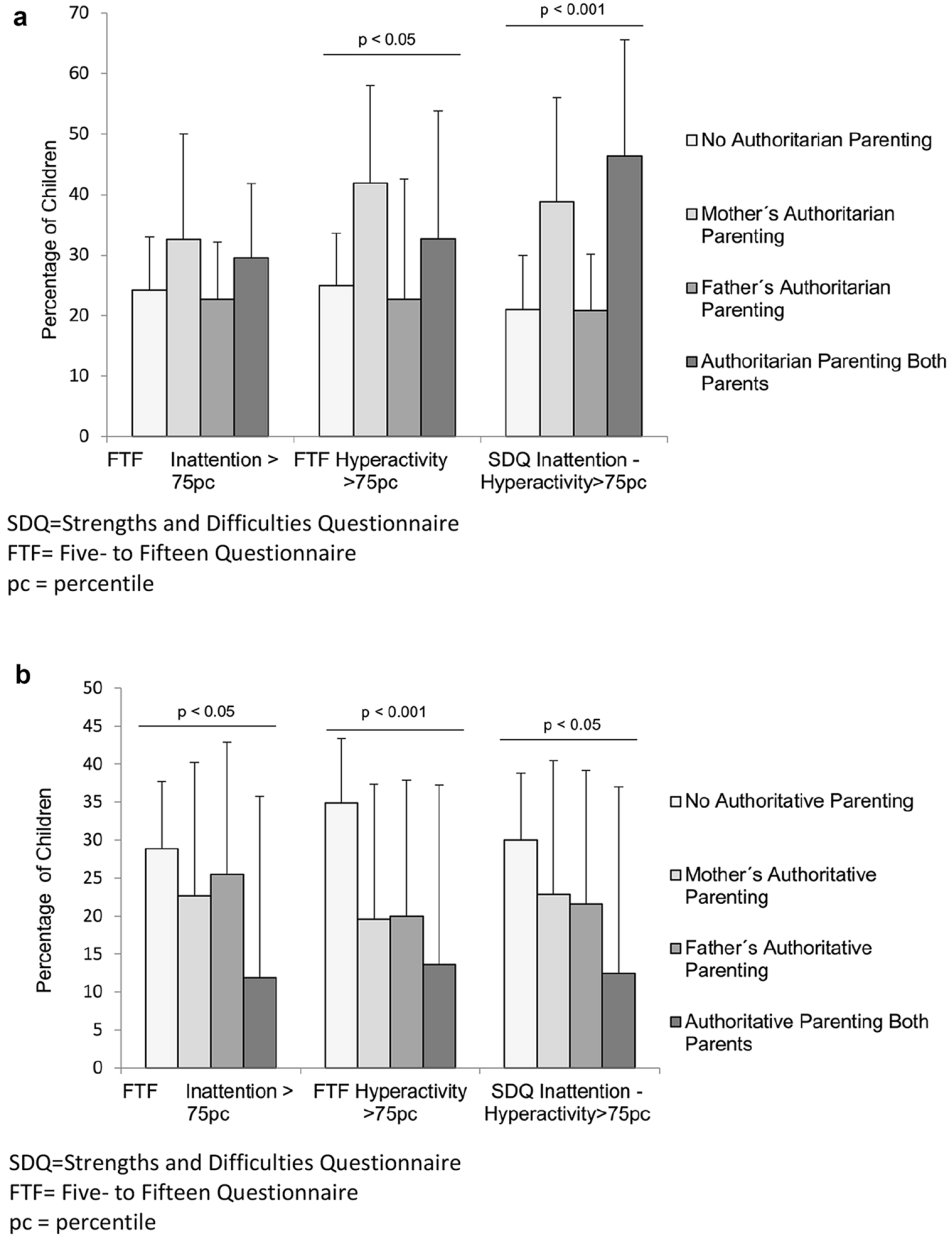
**a-b** Percentage of children having inattentive/hyperactive symptoms (score > 75pc) measured by FTF and SDQ in children at 5 years old, in terms of maternal and paternal authoritarian **a** and authoritative **b** parenting styles measured by PSDQ at 8 months Graphs a and b describe families with neither of the parents, only mother, only father, and both of the parents having an authoritarian **a** or authoritative **b** parenting style. Furthermore, both parent’s authoritative parenting style (B) is associated with lower scores in inattentive/hyperactive scale scores measured by both FTF and SDQ. Error bars represent 95% Confidence Interval of the proportion. P-values represent a significant difference between any of the four groups. Only significant differences are reported within the graphs. *SDQ* Strengths and Difficulties Questionnaire, *FTF* Five- to Fifteen Questionnaire, *pc* percentile, *SDQ* Strengths and Difficulties Questionnaire, *FTF* Five- to Fifteen Questionnaire

As Fig. 1 shows, the prevalence of inattentive and/or hyperactive symptoms in 5-year-old children was significantly related to parent-reported parenting styles (Fig. 1a-b). The highest percentage of children with elevated levels of inattention/hyperactivity symptoms appeared in families with both parents having an authoritarian parenting style while the maternal authoritarian parenting style was related to higher frequencies of hyperactivity symptoms, as measured by FTF (p < 0.05). Furthermore, 5-year-old children’s inattention/hyperactivity symptoms were less common in those families with authoritative compared to families with no authoritative parenting style (p < 0.05). Even having one parent (mother or father) with an authoritative parenting style appeared to decrease the risk of inattentive/hyperactive symptoms at 5 years of age.

Finally, when examining the cumulative effect of the most significant risk factors found in multivariate models, we observed that children with several risk factors (being a boy, maternal authoritarian parenting style, more negative family atmosphere, and persistent maternal depressive symptoms) had the highest risk (OR 8.40, CI 95% 3.17–22.30, p < 0.001) for having inattentive/hyperactive symptoms, as measured by SDQ at 5 years of age (Table 4a-b).

**Table 4:**
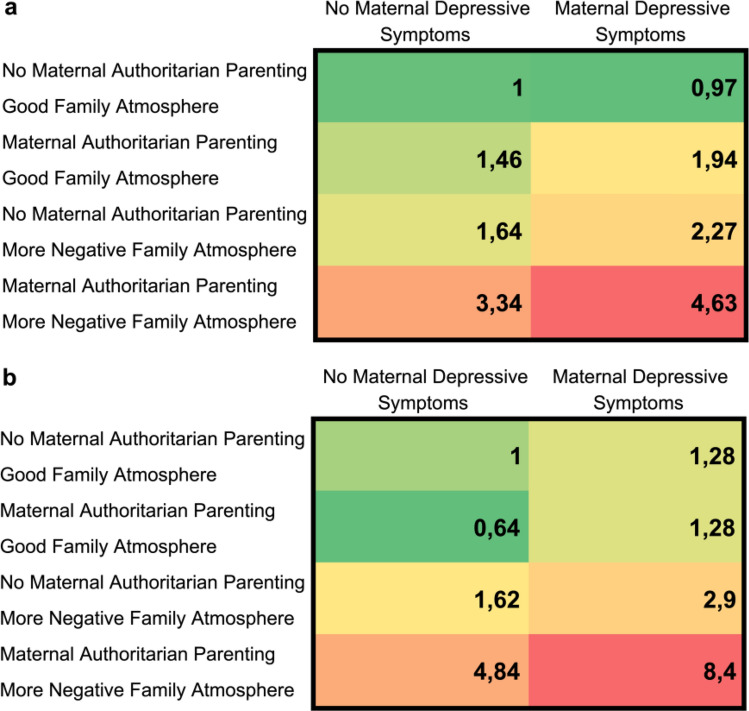
a-b Odds Ratios (OR) for **a** all the children and **b** for boys having inattentive/hyperactive symptoms measured by SDQ (n = 666) in 5-year-old children, in terms of maternal authoritarian parenting style, family atmosphere, and maternal depressive symptoms. Table a shows the cumulative effect of three risk factors on a child’s risk for having inattentive/hyperactive symptoms at 5 years of age. Children with several risk factors (being a boy, mother’s authoritarian parenting style, more negative family atmosphere, and persistent maternal depressive symptoms) had the highest risk for having inattentive/hyperactive symptoms (Table b)

## Discussion

The purpose of this study was to evaluate which prenatal and postnatal parent-reported risk factors best predict inattentive/hyperactive symptoms in 5-year-old children. This study is one of the few studies considering a wide range of potential parental risk factors during and after pregnancy in the development of children’s ADHD-related symptoms. Our study adds to previous research by examining both maternal and paternal early factors. In particular, the role of fathers’ mental health and early parenting style on children’s later inattentive/hyperactive symptoms are highlighted. Furthermore, the role of both parents having a similar parenting style and the cumulative effect of several risk factors on children’s ADHD symptomatology are also described.

According to our initial hypotheses, we found that gender (i.e. being a boy), persistent maternal depression from pregnancy until the child was 24 months old, parent-reported child’s shorter sleep duration at 3 months old, maternal authoritarian parenting style when the child was 8 months old, and low paternal income increased the risk for children’s inattentive and/or hyperactive symptoms at 5 years old. Moreover, persistent paternal depressive symptoms and a more negative family atmosphere reported by mothers when the child was 3 months old predicted the child’s ADHD symptomatology. Nevertheless, and contrary to our expectations, the paternal authoritarian parenting style was not related to children’s ADHD symptomatology, although an authoritative parenting style was associated with fewer inattentive and/or hyperactive symptoms. In our study, maternal advanced age was associated with hyperactive-impulsive symptoms. Contrary to our hypothesis, low birth weight and several prenatal factors such as parental smoking or alcohol use during pregnancy, parental ADHD symptoms, or parental education were not associated with children’s ADHD symptomatology at 5 years of age in the predictive models. Finally, we found that risk factors display a cumulative effect on children’s ADHD symptoms. The highest risk for a child to have inattentive/hyperactive symptoms at 5 years of age occurred in children with several risk factors (persistent maternal depressive symptoms from pregnancy to 24 months postnatally, more negative family atmosphere when the child was 3 months old, mother’s authoritarian parenting style when the child was 8 months old, and being a boy). Our study supports the hypothesis regarding the multifactorial aetiology of ADHD and emphasises the relevance of several postnatal environmental factors such as parental mental health, family atmosphere, and parenting strategies, in affecting the development of a child’s inattentive/hyperactive symptoms during infancy and the preschool period.

In our study, both maternal and paternal high depressive symptom levels were associated with children’s inattentive/hyperactive symptoms at the age of 5 years. Our results are consistent with previous studies reporting associations between prenatal [8, 18] and postnatal [8, 12, 17] parental depression and children’s later ADHD symptoms. Previous studies have, however, also reported contradictory findings, with no association between paternal prenatal [18] or postnatal [12] depressive symptoms when controlling for several confounding factors. It has been suggested that maternal prenatal depression is associated with an infant’s later outcomes by altering the mother’s HPA-axis activity [51]. An elevated maternal cortisol level may influence glucocorticoid action in the placenta and thereby create an adverse foetal environment [52]. Nevertheless, all mechanisms involved in the association between maternal depressive symptoms during and after pregnancy, and a child’s later outcomes are not yet well understood [51]. Other maternal factors such as maternal physical health and lifestyle during pregnancy, a child’s inherited genetic susceptibility to psychopathology, and several postnatal factors may also account for this association [11, 51, 53]. For example, depressed woman during pregnancy may engage in more unhealthy behaviours such as smoking or substance use during the pregnancy period. Furthermore, a child’s genetic susceptibility to depression could manifest as behavioural problems during the preschool period. We found similar associations between maternal and paternal high depressive symptom trajectories and children’s ADHD-related symptoms, suggesting that the effect is not solely mediated by intrauterine mechanisms, but that familial, environmental, and possibly genetic factors are additionally driving this association [53]. In our sample, the maternal and paternal depressive symptoms during and after pregnancy were relatively stable [46], and 7.4% of the mothers and 1.9% of the fathers reported constantly (from pregnancy to two years postpartum) depressive symptoms above the clinical threshold. Persistent maternal and paternal depressive symptoms from pregnancy to the postnatal period were associated with children’s inattentive/hyperactive symptoms, suggesting that postnatal depression also affects the development of children’s attentional control [8, 18]. In line with our results, a population-based study with 1779 mother–child dyads demonstrated that persistent maternal depression during and after pregnancy was related to children’s ADHD symptoms at preschool-age [8]. It should be noted, however, that they showed postnatal maternal depression to have an additive effect on prenatal depression. Another study with two separate birth cohorts found that both maternal and paternal depressive symptoms during pregnancy predicted children’s attentional problems at preschool-age, whereas associations between prenatal paternal depression and children’s attentional problems were substantially weaker and were overridden by maternal anxiety/depressive symptoms when the child was 3 years old [18]. Finally, it has been suggested that parental depressive symptoms may influence children’s later ADHD symptoms via their effect on parenting and especially via hostile parenting strategies [39]. Our study showed, however, that persistent parental depressive symptoms were independently related to children’s later inattentive/hyperactive symptoms even when several prenatal and postnatal factors, including different parenting strategies, were taken into account.

Another relevant finding in our study was, indeed, related to the parenting styles when the child was 8 months old. The maternal authoritarian parenting style was associated with more inattentive/hyperactive symptoms in 5-year-old children. In turn, authoritative parenting styles in both parents was related to fewer ADHD symptoms. Furthermore, the highest percentage of children with elevated levels of inattention/hyperactivity symptoms appeared in families with both parents having an authoritarian parenting style. By contrast, the lowest risk for ADHD symptoms was found in those families with both parents having an authoritative parenting style.

To the best of our knowledge, this is the first longitudinal study to report significant associations of both maternal and paternal parenting styles during the first year of a child’s life on the child’s later ADHD symptoms. The majority of the previous studies on the relationship between parenting and children’s later inattentive/hyperactive symptoms have focused on mothers. Some of these studies have reported the association between maternal sensitive parenting during infancy and/or early preschool age and fewer inattentive/hyperactive symptoms at preschool and school-age [37, 38, 40]. For instance, high levels of maternal responsiveness/warm behaviour, as measured by videotaped tasks, when the child was 2 years old predicted a greater increase in sustained attention in a laboratory task completed between 2–4.5 years old compared to mothers with no such behaviour [40]. Our study extends these previous findings by providing similar results regarding paternal consistent, supportive, and warm parenting since paternal authoritative parenting during infancy is also related to a lower risk of ADHD related symptoms. Furthermore, the effect of authoritative parenting by both of a child’s parents seemed to be additive, as children with two such parents had the lowest risk for ADHD symptomatology. Similarly, a study with 200 children living in low-income families reported that 2-year-old children with two supportive parents scored highest in cognitive tests at 5 years old, while children with two unsupportive parents scored lowest [54].

Additionally, and consistent with our hypothesis [22, 23], we found that a maternal authoritarian parenting style is associated with children’s later inattentive/hyperactive symptoms. Some previous studies suggest that harsh and/or intrusive parenting may be related to the disrupted development of children’s attentional control [9, 22, 23]. It has been reported that maternal intrusiveness at 6 months old or maternal overstimulating behaviour at 3.5 years old, as measured by questionnaires, are associated with teachers reporting more ADHD symptoms at 11 years old. Furthermore, a longitudinal Family Life Project study with 1173 children reported that early harsh-intrusive caregiving behaviours, measured at the ages of 6, 15, 24, and 36 months by 10-min video recordings of parent–child interactions, constitute a general risk factor for elevated ADHD symptomatology in early (3–6 years) and middle childhood (7–12 years), as well as both timepoints together [9]. Therefore, this evidence suggests that children may be particularly vulnerable to the effect of hostile parenting during infancy. Contrary to our hypothesis, however, a paternal authoritarian style did not predict children’s later inattentive/hyperactive symptoms in our study. Previous cross-sectional studies with school-aged children have reported that fathers’ inconsistent discipline or authoritarian control is related to children’s ADHD symptoms [32, 33]. The lack of this association in our study may reflect fathers’ tendency to spend less time with a child during the first year of its life compared to mothers with access to longer parental leaves.

Opposite findings have also been reported with no association between parenting and later symptoms of inattention/hyperactivity [12, 35]. For instance, in the Quebec Longitudinal Study of Child Development birth cohort study, self-reported coercive parenting or overprotection when children were 5 months old was not significantly associated with ADHD symptomatology when they reached 8 years old [12]. The study suggested that the absence of this association may be partly related to the weakness in the measures of parenting. Moreover, in another longitudinal twin study including 886 twin pairs aged 11–17 years, most associations between parent–child hostility at school age and children’s ADHD symptoms were accounted for by genetic factors. Boys’ ADHD symptoms impacted upon mother–child hostility both within and across time and there were no effects in the opposite direction. It should be noted, however, that the children included in the study were significantly older than in our study and, thus, hostility in the parent–child relationship may have different associations during infancy and later in childhood.

Regarding the association between early parenting and children’s later inattentive/hyperactive symptoms, we were interested to study whether the early parenting style would be related to the emergence of inattentive/hyperactive symptoms at preschool-age. This hypothesis was partly strengthened as we found consistent associations between early parenting and later symptoms of inattention/hyperactivity. Another explanation for this association would, however, be the possibility that negative parenting during infancy is evoked by child’s challenging behaviours (i.e. excessive crying, early sleep difficulties, feeding problems) related to the later diagnosis of ADHD [55]. Furthermore, it is also possible that parental ADHD symptoms predispose to negative parenting strategies with less positive parenting and more harsh/inconsistent parenting [56] and, thus, negative parenting and children’s inattentive/hyperactive symptoms may have a shared genetic background. While genetic factors (i.e. multiple known risk gene variants) make an important contribution to the development of ADHD [4, 13], environmental factors (e.g. negative parenting, parental depression, and negative family atmosphere) and/or child-specific factors (e.g. sleep difficulties) may exert the strongest influence over individuals with a particular genetic vulnerability [19, 57, 58]. For example, a previous review and meta-analysis supported the idea that multiple factors (a certain genotype, exposure to chemicals, and traumatic life events) increased the probability for a child’s ADHD diagnosis [19].

In our study, several prenatal factors were not associated with children’s later inattentive/hyperactive symptoms, as would have been expected based on previous studies [5–7, 12, 15–17]. For example, parental smoking or alcohol use during pregnancy was not significantly linked to the child’s later symptoms of inattention/hyperactivity. These results could be explained by a lack of power (i.e. the number of mothers smoking during pregnancy or using alcohol was rather small) or the genuine absence of an association. Among the prenatal factors, only paternal low income and the mother’s advanced age predicted the child’s later symptoms of inattention/hyperactivity. Contrary to previous studies [7, 12], we found maternal advanced age was a risk factor for children’s hyperactive symptoms, as measured by the FTF hyperactivity scale. Consistent findings have, however, been reported in a retrospective study with 58 children diagnosed with ADHD [15], where advanced maternal age was found to be associated with children’s ADHD symptoms at school age, possibly reflecting the sample characteristics (woman in the upper-middle socioeconomic class giving birth later in life). In addition, we found that a shorter sleep duration at 3 months was related to increased levels of inattention at 5 years old, which is in line with our previous report.[10].The specific role of sleep duration in the development of attentional control has been reported previously [10, 24–26], which suggests that the development of attentional control may be particularly vulnerable to the effects of sleep deprivation during infancy and early childhood, due to its complex and prolonged course of maturation during the preschool period [59].

Finally, we found a cumulative effect of several risk factors (i.e. being a boy, maternal persistent moderate/high depressive symptoms, maternal authoritarian parenting style, and a more negative family atmosphere) on children’s inattentive/hyperactive symptoms at 5 years old. Odds ratios for those children with two or more risk factors were substantially higher compared to the children with no risk factors or only one risk factor alone. Our results highlight that it would be crucial to identify high-risk children with several risk factors that might lead to the development of ADHD symptoms in later childhood already during pregnancy and the first year of a child’s life. Indeed, early interventions such as evidence-based parenting programs [60], effective treatment of parental depression [61], and treatment of a child’s early sleep difficulties [62] could prevent later symptoms of inattention/hyperactivity. In future studies, further examination of this genetic-environment interaction and intervention studies would be of great importance [4, 13].

The strengths of this study include the use of a broad range of prenatal and postnatal potential risk factors gathered from both parents, several measurement time-points of parental depressive symptoms from pregnancy to 24 months postnatally, and the assessment of ADHD-related symptoms with two questionnaires. However, there are also some limitations to consider when interpreting the present results. Firstly, the response rate when the child was 5 years old was relatively low (42.5%) and may have affected these results. The responding parents had a higher educational level and mothers reported fewer ADHD symptoms during pregnancy compared to the nonresponding mothers. As there are studies suggesting that lower parental education is associated with children’s inattentive/hyperactive symptoms [15, 16, 63], the children in our responding families may have been at lower risk of ADHD symptoms than those in the general population. Therefore, this bias may weaken the results in this study as there may have been a lower prevalence of children at higher risk of ADHD. Secondly, the families in this study were recruited at the 32nd pregnancy week and, therefore, the sample consisted of a high prevalence of children with full-term births and normal birth weights. This may also have affected the results by weakening them, as there are studies reporting the association between prematurity/low birth weight and later ADHD symptoms [6, 7, 9, 12]. Thirdly, we relied on parental reports when assessing the children’s inattentive/hyperactive symptoms. As we lacked information from other sources (teacher ratings or performance-based assessment) we do not know whether these parent-reported behaviours at home also manifest at, for example, day-care. Further, many parents of children with inattentive/hyperactive symptoms may also find themselves experiencing these symptoms [3, 4] and they may find it difficult to evaluate their child’s behaviour [64]. Furthermore, as parents suffering from depressive symptoms might see the world more negatively than healthy parents, it is also possible that parents with depressive symptoms overestimate their child´s behaviour problems due to their own mental health issues. Future studies on the topic should aim to confirm these findings with a comprehensive evaluation of the children at school-age (teacher ratings, parent ratings, diagnostic evaluation). Fourthly, the majority of the risk factors were self-reported, and parents may have underreported their psychiatric symptoms and/or substance use during pregnancy, possibly leading to an underestimation of these effects. Finally, the Cronbach’s Alpha value for permissive parenting was rather low (α = 0.53) for both parents and may explain the lack of association between permissive parenting and inattentive/hyperactive symptoms or, alternatively, describe the inconsistency in the parenting style within the responders that belonged to this category.

## Summary

The aim of our study was to evaluate which prenatal and postnatal parent-reported risk factors associated with inattentive/hyperactive symptoms in 5-year-old children. Our study adds to previous research by examining both maternal and paternal early factors. We found that persistent parental depressive symptoms during and after pregnancy, as well as maternal authoritarian parenting, more negative family atmosphere, and a child’s short sleep duration are related to the child’s later ADHD symptomatology. Furthermore, authoritative parenting styles in both parents were related to fewer ADHD symptoms. The results of this study highlight the importance of parental mental health and family atmosphere, and the role of early parenting strategies and a child’s sleep duration on the developmental course of the child’s later inattentive/hyperactive symptoms. As children’s early development is a critical period of vulnerability, we emphasise the need for early screening for several risk factors of ADHD-related symptoms, as preventive interventions for parental mental health and supportive parenting training programs may benefit not only parents but also their children’s development.
